# Sudan's catastrophe: the role of changing dynamics of food and power in the Gezira agricultural scheme

**DOI:** 10.1111/disa.12663

**Published:** 2024-10-30

**Authors:** Tamer Abd Elkreem, Susanne Jaspars

**Affiliations:** ^1^ Department of Anthropology University of Khartoum Sudan; ^2^ SOAS Food Studies Centre University of London United Kingdom

**Keywords:** food insecurity, neoliberalism, political economy, Sudan/Gezira agricultural scheme

## Abstract

This article explores the role of historical, political, and economic processes in understanding war and famine in Sudan after 2023. The focus is on Al‐Gezira, the site of Sudan's largest agricultural scheme. Using ethnography, interviews, and document reviews, the study analyses the Gezira irrigation project in three phases. First, the 1980s and 1990s, when patronage politics dominated its management. Second, the neoliberal strategies of the Gezira Scheme Act of 2005, which enabled Islamist profiteering while increasing vulnerability among farmers and labourers and tensions between them. Third, post 2018, when political movements used evidence of the scheme's deterioration to call for revolution, but once achieved, previous tensions grew and have been manipulated during the war. Sudan provides an example of how decades of war and neoliberal economic strategies have led to a deeply‐rooted, violent, and extractive political economy. This has been to the benefit of business and elites, leaving many to a life of precarity, exploitation, and hunger.

## 1 INTRODUCTION

The question of why a country like Sudan, bestowed with enormous agricultural resources, cannot feed itself becomes more relevant during times of crisis. Such a question can only be answered with an understanding of Sudan's political economy and how historical processes have influenced the food security of different population groups in Sudan. This article focuses particularly on Al‐Gezira state, the site of Sudan's biggest agricultural scheme, and on why understanding historical political and economic processes is important for analysing how some gain power and others become vulnerable to food insecurity. This is especially pertinent in the context of war and famine in 2024.

Al‐Gezira is unique in Sudan because it has historically been where governments have prioritised the country's development, and where people from the peripheries (Darfur, Kordofan, and the South before secession) sought work when faced with the risk of famine. Al‐Gezira hosts the biggest irrigation project in the country, comprising 42 per cent of Sudan's irrigated area, and until recently, provided a livelihood for almost two million tenant farmers and seasonal labourers (Eldaw, 2004). Since December 2023, it has also become one of the largest battlegrounds following the descent into nationwide war.

Sudan has a long history of food insecurity and famine. From colonial times, wealth and power have been concentrated in the hands of an interconnected political and economic elite, composed of religious and traditional leaders, ministers, and businessmen, mostly from Arab tribes in the country's riverine heartland (Niblock, 1987). Development policies have concentrated on the introduction of modern commercial agriculture in central Sudan, in places like Al‐Gezira, which are in turn dependent on labour from impoverished famine‐ and war‐stricken regions such as Darfur and the South (O'Brien, 1983; Maxwell, 1991). In the past three decades, a military–security–industrial complex has controlled the country's key resources, including agriculture, trade, banking, fuel, and gold (Gallopin, 2020). Those who form part of this complex, which may encompass government officers, businessmen, and army and militia forces, have benefited enormously. In parallel, populations in Sudan's peripheries have suffered food insecurity and famine, due to political and economic marginalisation as well as war, drought, and economic mismanagement. War and famine themselves have yielded benefits for central Sudan's elite (Keen, 1994), and the ways in which production, markets, and aid are manipulated to the advantage of powerful (sometimes armed) groups remains highly relevant today. The impoverishment and political marginalisation of rural populations have led to rebellions (for example, in the South and in Darfur) and enabled the use of a militia strategy of warfare by Sudan's former government. Impoverished (Arab) nomadic populations have formed the backbone of the militia, some members of which later evolved into the Rapid Support Forces (RSF).

War, corruption, and economic crisis led to Sudan's popular uprising, the ousting of President Omar Al‐Bashir in April 2019, and a transitional government later that year. Claiming participation in Al‐Bashir's overthrow, the RSF became part of the power‐sharing arrangement between civilian and military establishments. However, the potential loss of control by military and security actors over key economic resources led to, first, the military coup in October 2021, and then the war between the Sudan Armed Forces (SAF) and the RSF in April 2023. As such, the war that has engulfed Sudan since April 2023 can be seen as an intensification of its extractive political economy.

In this article we build on the long history of food security and famine research in Sudan, in particular that of Keen (1994) and Duffield (1990, 1994) to analyse changing dynamics of food and power in the Gezira agricultural scheme up to mid‐2024. We examine the political and economic processes by which food insecurity and famine are produced in some groups, and that enable others to benefit. In other words, the maintenance of power by asset‐stripping of politically weak populations. Market manipulation, state appropriation of land and other assets, and raiding and theft have been a key part of these processes. Aid, through its selective provision or denial, has become significant too in this respect (Jaspars, 2018). This is similar to a relational approach to poverty analysis (Mosse, 2010), which we use to analyse how multidimensional forms of power interplay to produce, maintain, and deepen structural causes of poverty or, in our case, food insecurity. These forms of power in Al‐Gezira range from economic exploitation by crony capitalists, to discrimination, to the agenda‐setting powers of the Gezira Scheme Act of 2005 (Government of Sudan, 2005), to the current violence and aid manipulation during the war.

The paper investigates how this has affected the food security of farmers and labourers in particular, as well as how exploited and excluded people have resisted these processes and how acts of resistance themselves have been manipulated at different historical points. Such analysis is rarely conducted as part of food security assessments. We also build on studies of the nature of warfare in Sudan, and how ethnic divisions have been exploited for military and political ends (Suleiman, 2006). The article focuses specifically on changing power relations between state, private sector, tenant farmers, and labourers in the Gezira agricultural scheme. In studying the latter, we spotlight farming and farmers rather than pastoralism or nomadic populations. While Al‐Gezira was rangeland before the scheme's establishment, and some pastoralists whose rangeland was taken became project tenants or labourers (Abdelkarim, 1986; Blench, 1987), our main aim is to examine the changing power relations within the Gezira agricultural scheme—its board, tenant farmers, and labourers.

In terms of methods, we build on six months of intensive ethnographic fieldwork in Gezira state in 2017, focusing especially on the effects of the Gezira Scheme Act of 2005 (Tamer Abd Elkreem), past research on the political economy of food and aid (Susanne Jaspars), and exploratory work for our current project.^1^ For the latter, we are collecting secondary and primary data through interviews and observations by research assistants on the ground who closely follow the post‐war dynamics and their food security implications.

The article starts with an overview of the current situation in Sudan and Gezira state, including the effects of the war on civilians, reports of food insecurity, and probable famine. Subsequent sections analyse three recent periods chosen to reflect key changes in the Gezira scheme in terms of political economy, governance, and power relations. First, a brief look at Al‐Gezira from the 1980s to the late 1990s, during which (following Al‐Bashir's Islamist coup in 1989) a combination of privatisation and governance through patronage meant that those close to the regime benefited. Second, the effects of the Gezira Scheme Act of 2005, which introduced rehabilitation policies that were more aggressively neoliberal—with respect to the withdrawal of the state, yielding further benefits for those close to the Islamist regime, and which debilitated the scheme's production capacity (Abdul Hadi, 2011). A key aspect of this phase was the tension created between the State and its associated businesses on the one hand, and farmers and labourers on the other. The third period is post revolution (from 2018) in which the Gezira scheme illustrates key events of the past six years. Its mismanagement was used by the opposition to illustrate the corruption of Al‐Bashir's regime and thus contributed to the revolution. In addition, the failure to address the tenants' and labourers' grievances contributed to the 2021 coup; and after April 2023, tensions and inequalities were manipulated as part of the political economy of war. Large numbers of displaced people arrived in Al‐Gezira from the capital of Sudan, Khartoum, following April 2023; and many fled after it was taken by the RSF in December of that year. Using the analysis in this paper, we argue that the current war, humanitarian crisis, and probable famine, can only be fully understood in relation to Sudan's past political economy and that this knowledge needs to inform the response.

## 2 WAR AND FAMINE IN 2024, INCLUDING IN Al‐GEZIRA

The post‐April 2023 war has made the already serious political, economic, and humanitarian crisis in Sudan much more severe. This section provides an overview of the crisis in early 2024. War has increased the number of people in need of humanitarian assistance from 15 million in 2023 to 24.8 million in 2024, almost one‐half of the country's population (UN OCHA, 2022, 2024). Eight million people have been newly displaced as a result of warfare, with almost two million fleeing the country (to Egypt, Chad, Uganda, South Sudan, Ethiopia, Libya, and the Central African Republic) (UN OCHA, 2024). The combined effect of displacement and disruption of production, trade, markets, the financial system, and connectivity, means that 18 million people in Sudan faced acute hunger in 2023 during Sudan's traditional harvest period (WFP, 2023a). At the same time, the World Food Programme (WFP) was only able to reach 1 in 10 of the people experiencing emergency levels of hunger (WFP, 2024).

As soon as the war started in Khartoum, basic services such as drinking water, electricity, hospital services, life‐saving drugs, fuel supplies, communications networks, and banks, ceased to function. Food imports, trade, and transport across the country were severely disrupted within Sudan, and grain prices rose steeply (FEWS NET, 2023). International aid organisations fled and/or faced extreme challenges in being able to respond. Financial transfers from relatives and friends were hindered by the destruction of Sudan's financial system, the closure or destruction of banks, and the interruption of communication networks (Jaspars and Abd Elkreem, 2023). Many people fled from Khartoum to Al‐Gezira, which became the second biggest destination for people fleeing the capital and other war‐torn areas, hosting more than 300,000 displaced persons and 50,000 refugees (Shabaka and Naiel, 2023). In Darfur, the conflict worsened from April 2023^2^: large‐scale violence, destruction, killing, rape, and displacement led human rights organisations to compare it with the genocide of 2003–04 (Human Rights Watch, 2023). Food insecurity deteriorated accordingly. In June 2023, the highest level of food insecurity was in Khartoum, Darfur, Kordofan, and Blue Nile (FEWS NET, 2023); by early 2024, this also included Al‐Gezira (see below). Whereas peripheral states like Darfur historically experienced frequent food crises and famine, Al‐Gezira state had been Sudan's ‘breadbasket’—as shown by its high food security status in recent WFP (2022, 2023b) surveys. Moreover, as revealed in the next section, labour opportunities provided by the Gezira scheme were used by people facing the risk of famine in places like Darfur.

In December 2023, the RSF invaded Wad Medani, the capital of Al‐Gezira state, and the army quickly withdrew. They destroyed and looted markets, banks, people's properties and food stores, the seed bank, and the warehouses of humanitarian aid organisations (FEWS NET, 2024a; Nordling, 2024). Sexual violence was employed as a war strategy (Zaman, 2024). An estimated 500,000 people fled from Al‐Gezira, 46 per cent of whom had fled earlier from Khartoum (FEWS NET, 2024a). Any attempts by citizens to resist lootings and sexual assaults were (brutally) met with direct shooting and killing (Radio Dabanga, 2024). Furthermore, those who wanted to acquire weapons to defend themselves could only do so by joining the RSF, drawing them into the war.^3^ Others have reported forced recruitment into the RSF under threat of starvation, by withholding food (Munsi et al., 2024). Aid organisations, which had only just established themselves in Al‐Gezira, also left.

The attack on Al‐Gezira interrupted the sorghum and millet harvest and disrupted winter wheat cultivation (FEWS NET, 2024b). The 2023–24 harvest was estimated to be about 40 per cent lower than usual in the country overall, and wheat and sorghum production in Al‐Gezira was almost 50 per cent lower (FAO, 2024; Hoffmann, 2024). Agricultural credit decreased, the cost of inputs increased (such as seeds and fertiliser), fuel was in short supply, and the irrigation infrastructure was destroyed (FAO, 2024). Traders moved stocks out of the main markets owing to fear of looting, and cross‐country trade routes have been even more affected as Al‐Gezira is at the centre of them (FEWS NET, 2024b). A total communications blackout in February–March 2024 hindered trade, financial transfers, and aid (FEWS NET, 2024b). Even local Emergency Response Rooms (ERRs), which had been able to provide aid when international organisations could not, were unable to function without financial donations, and hence 200 out of 300 community kitchens in Sudan closed in February (FEWS NET, 2024b). For the first time, Gezira has become one of Sudan's food‐insecure war‐torn states. The main food security classification system has issued warnings of famine (IPC, 2024).

To analyse the nature and dynamics of this food insecurity, and its effect on the country overall, it is necessary to understand the history of the Gezira agricultural scheme and how it became emblematic of the country's extractive political economy. This political economy is based on patronage, privatisation, Islamist take‐overs, and expanding inequalities. This is discussed in the following sections.

## 3 THE HISTORY AND SIGNIFICANCE OF THE GEZIRA SCHEME IN SUDAN

This section provides an introduction to the Gezira agricultural scheme and its decline in the 1990s, which provided justification for privatisation and its assets ending up in the hands of those close to the Islamist regime. This is not to say, though, that there was no decline before the 1990s. Low yields and insufficient tenancy income led young tenants to seek job opportunities in other sectors or to migrate to Arab oil‐producing countries from the 1970s (Abdelkarim, 1986). However, the nature and scale of the scheme's deterioration from the 1990s is different, as we examine in the subsequent sections.

The Gezira project was first established in the 1920s by the colonial government to provide cotton for the British textile industry (Gaitskell, 1959). It continued to serve as the economic backbone of post‐colonial Sudan, constituting 60 per cent of export revenue in the 1970s, and providing employment for nearly two million tenant farmers, seasonal labourers, employees, and administrators (O'Brien, 1981). It remains one of the largest irrigated agricultural schemes in the world (FAO, 2024). As one of Sudan's most strategic economic projects, it has been subjected to the ideological fluctuations of different political regimes. These have ranged from military dictatorship, sectarian‐based democracy, and socialist governments to the self‐empowering Islamist regime, and finally to the 2018 revolutionary government, military coup, and war. National strategies have been closely linked to globalising tendencies (Barnett, 1977), including the shift from developmental state to neoliberal ideologies characterised by privatisation, free markets, and trade liberalisation. By 2009, the Ministry of Agriculture and Forestry's delegated study team considered the scheme to be a ‘a total failure’ (Salman, 2010). Key to its deterioration were changes in the tripartite sharecropping relations between the Sudan government, the Gezira Board, and tenant farmers. This section starts with a description of the sharecropping system, how it functioned in the 1970s, and the changes brought about by structural adjustment and Islamist government strategies in the late 1980s and early 1990s.

The cornerstone of the Gezira management system was a tenancy agreement defining the rights and duties of each of the three stakeholders. For a long time, it concerned only cotton production, but from the 1990s also included wheat. Other crops such as groundnuts, sorghum, and fodder were exclusively the responsibility of the farmers. The government supplied the water and finance needed for the production of cotton, the Gezira Board provided central management and mechanised work, and the tenant farmers were responsible for other cultivation processes, irrigation cleaning, and picking of cotton. After the deduction of the costs, the income was divided as follows: 40 per cent for the tenant farmers; 40 per cent for the government; and 20 per cent for the Gezira Board (Salman, 2010).

As cotton picking is a labour‐demanding activity and could not be handled by the tenant farmers and their families alone, the scheme attracted thousands of seasonal workers. These labourers came mainly, but not exclusively, from West Africa, Darfur, and the Nuba Mountains, and included Arab pastoralists from both Blue Nile and White Nile states. The ‘tripartite’ tenancy agreement excluded seasonal labourers, who were thus more vulnerable to exploitation (Abdelkarim, 1986). The huge expansion of mechanised agriculture in the 1960s, and the transformation of the economy, meant that by the mid‐1970s more than one million people migrated annually to central and eastern Sudan in search of work (Duffield, 1990). While seasonal labour was at first actively recruited by the scheme's management, labour migration accelerated with the food crises, war, and famines from the 1980s onwards (African Rights, 1997).

Seasonal labourers gradually settled and brought their families, living in camps that were scattered across the scheme, but maintaining their linguistic, ethnic, and cultural uniqueness. Although some of these camps are as old as the farmers' villages, they still lack basic services. The seasonal labourers were not allowed to own land for cultivation or settlement, and nor were they permitted to establish a representative body that speaks for them (Salman, 2010).

The Gezira Board had several departments—administration, agricultural engineering, cotton processing, finance, mechanical and civil engineering, railways, seed propagation, and social development—as well as workshops for agricultural machineries. This large bureaucratic body employed thousands of administrators, engineers, researchers, block and field inspectors, and workers. The scheme's assets, such as heavy agricultural machineries, tractors, harvesters, the railway, research centres, and accommodation for senior clerks and administrators, equipped the Board with enormous capacity. In its early days, it regularly cleaned and maintained 300 kilometres of canals, and closely monitored them to deliver water on time and in the required volume. A unified crop rotation system, well‐recorded agricultural data, a functioning telecommunications system, and highly standardised agricultural activities contributed to this efficiency. This highly centralised management system depended on thousands of inspectors who gave instructions to farmers, kept records, received complaints, and organised payments and loans. It also provided inputs for land preparation, seeds, fertiliser, pesticides, and all other agricultural services. Given the size of the Gezira Board, it had the bargaining power to sell the produce and buy inputs at the best price possible.

By the 1980s, however, the scheme was subjected to neoliberal policies encouraged by the World Bank and the International Monetary Fund (IMF), including privatisation (Salman, 2010). At the same time, a failed military coup marked a shift from a socialist regime to a capitalist regime closely linked to the country's Islamists. The previously strong Gezira scheme Farmers' Union was replaced by one loyal to the (Gaafar Nimeiri) government. Loyalty was gained through incentives such as preferential treatment when importing agricultural machineries, which allowed union members to accumulate wealth and to create further patronage networks. These political processes gradually silenced farmer's voices and manufactured their consent.

In 1989, President Al‐Bashir came to power in an Islamist‐backed military coup. His regime adopted a project of national food self‐sufficiency and policies. The National Economic Salvation Program and the Comprehensive National Strategy of 1990 and 1992, respectively, included increased investment in agriculture (expanding the irrigated sector), the liberalisation of trade, the privatisation of state assets, the removal of food subsidies, and the establishment of a national strategic reserve (Elbashir and Ahmed, 2006).

In practice, this meant self‐empowerment (*tamkeen*) through the firing of political opponents and the hiring of loyalists through a policy called *Al saalih al ‘am* (public good). This compromised the quality of the civil service across the whole nation. The Gezira Board's bureaucratic machinery was one of the Islamists first targets of self‐empowerment, which compromised the Board's quality of expertise, its accountability, and the overall performance of administration (Abdul Hadi, 2011). Water canals were no longer routinely cleaned and the inspectorate system was less rigorous; as a result, farmers were not getting timely irrigation for their crops.^4^

The Central Bank of Sudan withdrew its financing for the scheme (Eldaw, 2004), and less accountable channelling of funds to the Gezira Board, from other banks, led to lower quality agricultural services. Services reached the farms when the appropriate time of the season had already passed.^5^ The price of agricultural inputs increased, leading to declining yields and profitability. Farmers complained about the malfunctioning Gezira Board and accumulated debts.

By the mid‐1990s, irrigation intensity declined by 50 per cent. The scheme's debts led to inadequate funding for operations and maintenance, the silting of canals, and reduced crop production. Income from farming was no longer sufficient for 130,000 farmer households. Consequently, they started to engage in sharecropping and livestock production to supplement their income; many abandoned farming altogether (Salman, 2010). The living conditions of seasonal labourers got worse, but most stayed in the scheme owing to a lack of alternatives. These deteriorating conditions provided the justification for the Gezira Scheme Act of 2005, discussed in the next section.

## 4 THE 2005 GEZIRA SCHEME ACT

The gradual degradation of the scheme was problematised by ruling Islamists and their patronage networks to introduce the reforms of the Gezira Scheme Act of 2005. Officially, the Act aimed to use production resources and potential to improve the economic and social standards of farmers and their employees (Government of Sudan, 2005). However, its implementation led to the withdrawal of the state, including the replacement of the Gezira Board with Islamists' private companies, the liquidation of publicly‐owned scheme assets, and the transferal of irrigation responsibility from the government to farmers through Water Users Associations (WUA). This section sheds an ethnographic light on what governed the scheme's performance from below and situates conflicting interests and visions within their wider sociopolitical contexts of Sudanese state–society relations. It argues that in practice, the 2005 Act increased vulnerability to food insecurity for both farmers and labourers, while increasing the wealth of prominent Islamists and their cronies. The section starts with the national political and economic setting, followed by the nature of the reforms introduced, how they increased the vulnerability of farmers and labourers and tensions between them, and how traders, businessmen, and wealthy farmers benefited.

The 2005 Act was introduced at a time of peak economic growth in Sudan, because of oil revenue and the Comprehensive Peace Agreement following the war in southern Sudan. Much of this revenue was used to maintain political loyalties and was spent on defence and national security (Patey, 2010; de Waal, 2019). Almost all federal ministers pushed for privatisation and unleashing market potentiality. Islamist entrepreneurs seduced Gulf Arab financiers by offering them a magical combination of free water, cheap labour, and ‘easy’ land. This established regional connections through which money poured into Sudan's core areas (Verhoeven, 2011). Many Islamist leaders progressively took ownership of big companies, private universities, private hospitals, import–export companies, construction companies, and heavy machineries. In Al‐Gezira, lower‐level regime loyalists also pushed for market‐oriented reforms because they benefited from preferential access to loans and exchange rates for imports and trading arrangements. It was also, of course, a time of war, humanitarian crisis, and genocide in Darfur (Prunier, 2008), leading to forced displacement and an increased supply of cheap and exploitable labour in Al‐Gezira and elsewhere.

The Islamo‐patronage capitalists adopted a new reformist discourse towards the Gezira scheme's rehabilitation. The Gezira Board was described in this discourse as a ‘traditional way of management’ that does not tap into farmers' accumulated experiences and entrepreneurship. For instance, one of the proponents of the 2005 Act, a relative of the President and a billionaire who owns an agricultural services company, argued:

*The traditional, rigid, disciplinary, paternalistic ways of management no longer suit the ambitious, educated and fully experienced Gezira farmers who are eager to take full responsibility of all agricultural processes and make the best out of their fields*.^6^

The Act's reforms were implemented via a combination of liquidation of the scheme's assets through government committees and transferal of irrigation responsibility to the WUAs. The committees sold assets such as agricultural engineering workshops and its machineries (such as harvesters, tractors, and the railway) (Abdul Hadi, 2011). Hundreds of buildings (administration facilities, houses, research centres, and stores, for instance) had their furniture, windows, and roofs stolen. Most of the farmers interviewed used expressions like ‘they have stolen’ and ‘the scheme is ruined’. One said: ‘the government destroyed our muscles and told us you are free to move’. In 2011, the government issued tenders to establish private companies for integrated agricultural services. The establishment of these companies mostly benefited local leaders of the ruling party, most of whom were also leaders of a government‐created loyalist Farmers' Union. Twenty‐three companies were registered for integrated agricultural services, of which the Farmers' Union owned 18. To transfer irrigation management responsibilities, more than 1,500 WUAs (one for each minor canal) were established in 2007. Their responsibilities were carrying out farm irrigation and management, including the collection of fees and weed cleaning (Goelnitz and Al‐Saidi, 2020).

The proponents of the 2005 Act described the transfer of responsibilities to farmers as giving them the freedom to choose what to cultivate, to enter into contracts, to manage inputs and output, and to sell their land use rights. But it had a devastating effect on the scheme. In the course of one year, the staff of the Gezira Board was reduced from more than 7,000 to just 74 (including administrators, engineers, researchers, and inspectors). In practice, the WUAs were neither freely elected nor trusted by the farmers, thus failed to fill the huge gap left by the abolition of the Gezira Board. Instead, most farmers individually pursued their financing, land preparations, and other farming decisions. Even farmers who irrigated using the same canal no longer cultivated in a unified cropping pattern. The absence of a governing body responsible for overall coordination created great difficulties for irrigation and water‐sharing between farmers. For example, in thousands of major canals, the doors that controlled the quantity and flow of water were completely removed or replaced by a pipe.^7^

Furthermore, the Islamist regime silenced farmers' voices by preventing the free election of union members and by establishing parallel bodies from among its loyalists and acknowledging them as the Farmers' Union or farmers' leaders or representatives. The farmers who perceived these loyalist unions as a threat set up their own Farmers' Alliances which resisted Islamist policies. Later, the scheme's gradual degradation produced more anti‐regime bodies, such as the Farmers' Sons Union, the United Canabi Congress, and Al‐Gezira‐based resistance committees (see the next section). Confusion about these competing actors is evidenced, for example, in a World Bank report of 2000, which stated that: ‘While there continues to be resistance to change from those who would lose as a result of any institutional and managerial changes, there is a strong commitment by the Government, the SGB [Sudan Gezira Board] and the Farmers’ Union to the reform of the Gezira Scheme’ (World Bank, 2000, p. iii). The regime‐created Farmers' Union was instrumentalised as “farmers” commitment to the reform. Once the reform, or privatisation, had been realised, these institutions were no longer needed, and the government banned unions.

The 2005 Act and associated strategies left tenant farmers vulnerable to market exploitation, overpriced inputs, and underpriced output. It created space for brokers, who profited from the increased cost of agricultural inputs such as seeds, pesticides, and fertilisers, and farmers no longer enjoyed collective bargaining power. Most of the privatised service‐providing companies were owned by powerful Islamists connected to the state. Farmers felt that the scheme was targeted by global capitalists with government and Islamist capitalists as their agents. As discussed later, farmers responded to the destruction of the scheme with varying degrees of activism.

As farming became less profitable, most tenant farmers increasingly engaged in off‐farm activities, migrated to cities in Sudan or abroad, worked in the informal sector, such as petty trading, or sublet their farms. In a group discussion in 2017, farmers estimated that just 30 per cent of their livelihood depended on agriculture; the remainder was 30 per cent on migration, 20 per cent on informal activities such as petty trade, 10 per cent on formal employment (such as civil service and business), and 10 per cent on other miscellaneous endeavours. Some of the participants argued that those who migrated should be called internally displaced persons (IDPs). Another strategy was to enter into sharecropping agreements with labourers or camp dwellers (locally known as *Canabi*) who were previously only employed seasonally. This new agreement shared costs and yields, based on an understanding that tenant farmers' land equals the workers' labour. However, neither were comfortable with this arrangement. The tenants found it degrading to enter into arrangements with people they previously employed as seasonal workers. They consider themselves to be superior indigenous Arabs and the camp dwellers to be inferior Africans, revealing racist overtones—behind closed doors many of them would label *Canabi* dwellers as slaves (*beed*); a perception associated with the heritage of slavery (McLoughlin, 1962). The camp dwellers considered it unfair that they had to do this work because they lacked other opportunities. Moreover, the difference in services between the tenant farmers' villages, which had access to all basic services, and the labourers' camps, which lacked all of them, was a source of resentment.

In 2013, some of the educated youths and leaders among the labourers established the United Canabi Congress, in close collaboration with their fellow seasonal workers in other irrigated and rain‐fed agricultural schemes. Its main purpose was to fight against their sociocultural, economic, and political discrimination: being treated as second‐class citizens in their own country and living in undignified conditions without electricity, running water, schools, clinics, or even just a piece of land on which to build their houses. Although both tenant farmers and labourers were negatively affected by the deterioration of the Gezira scheme, Farmers' Alliances and the United Canabi Congress never effectively collaborated in fighting those who caused it.

Those who benefited from the 2005 Act included traders, company owners, service providers, and wealthy farmers. The argument of these winners was that small farmers lost out because they are lazy. The representation of farmers as lazy, incompetent, and leisured is deeply rooted in the genealogy of disciplinary colonial power since the scheme's establishment, as a way of justifying reforms and creating a highly controlled peasantry (Bernal, 1997).

The winners proudly took one of us to see their flourishing farms. Those who benefited the most were members of the Islamised Farmers' Union who were given the chance to borrow large sums of money from the agricultural banks to establish service‐providing companies. Their neighbours observed that these members became billionaires within the span of a few years. However, one company owner argued in an interview:

*There is nothing strange in the way we owned these companies, as it required a risk‐taking individual to borrow all these amounts of money and many of those who were offered the chance refused. We have taken the risk and started to work very well with the farmers, but we were targeted by the old GB [Gezira Board] administrators who are still fighting to restore the old system. They spread rumours that we were given these companies to work for them and some farmers feel entitled not to pay for us or complain at our prices though we deliver the cheapest rate. Now the government is committing a big mistake and gradually strengthening the Gezira Board administration again. Few difficulties we have faced during the last years is normal because farmers were given too much freedom to handle*.

As this quote shows, by 2011, the 2005 Act was rejected not only by farmers and activists but also by former powerful Islamist technocrats. This should be understood against the background of a regime that lost much of its oil revenues after the cessation of South Sudan (in 2011) and thus had much less access to resources to buy political loyalty. The regime gradually losing its grip manifested in the disintegration of homogenised patronage alliances, coupled with mounting resistance from below. In 2014, the government made amendments to the Act (Sudan Council of Ministers, 2013), including the ‘[replacement] of the WUAs with agricultural cooperatives (Farmer Unions), while the scheme's maintenance and management of the irrigation (including water allocation) was transferred to the Ministry of Agriculture and Irrigation’ (Goelnitz and Al‐Saidi, 2020, p. 5); this signalled the gradual return of the state that the company owner above is complaining about.

Given the deepening economic crisis, reversing the aggressive neoliberalisation was entertained at the highest level. For instance, the former Federal Minister of Agriculture who also served as the federal Minister of Finance, Ibrahim Mahmoud, gave the following account in response to Gezira farmers' complaints about the malfunctioning of the 2005 Act:

*The state is ready to enact any policy that benefits the farmers. When the farmers win the state wins and the citizens who are waiting [on] the farmers also win. Wheat has a huge market, and we want our farmers to make use of this market. . . . Therefore, the price we have announced for the ton of wheat is double the international price. . . . We have agreed with the President that any part that curtails the production from 2005 Gezira Act will be changed*.^8^

However, the 2014 amendments were too little too late. The resistance was getting more radicalised and gaining momentum, including in Al‐Gezira, where the Farmers' Alliance effectively coordinated with other mushrooming revolutionary bodies to overthrow the regime, as shown in the next section.

## 5 RESISTING AND RETURNING TO NEOLIBERAL STRATEGIES: REVOLUTION, TRANSITIONAL GOVERNMENT, AND WAR

This section analyses the period from 2018, in terms of the power dynamics within the Gezira scheme as a reflection of those in the country overall. This includes, first, the process that led to the 2018 revolution, and the role of the Gezira scheme. Second, it looks at the heightened tensions following the half‐successful revolution that led to compromised power‐sharing with the army and security sector within a transitional government that was unable to meet the demands of civilian movements—this paved the way for the 2021 military coup. Third, it explores events from April 2023, when the coup leaders from the SAF and the RSF turned against each other, and, in December, the RSF invaded Al‐Gezira, taking the long‐standing extractive violent political economy to a previously unmatched barbaric level.

### 5.1 How the failure of the Gezira scheme contributed to the revolution

Shortly before the revolution, the failure of the Al‐Bashir government to make the best use of the Gezira agricultural scheme was instrumentalised by different anti‐regime movements. Its deterioration stood as one of the biggest arguments against the Islamists' policies, corruption, and kleptocracy. Well‐organised new youth‐led activist bodies such as the Gezira Sons' Union and resistance committees added to older ones such as the Farmers' Alliance and the United Canabi Congress, and were supported by national opposition political parties. These revolutionary organisations circulated images of ruined infrastructure, comparing before and after the implementation of the 2005 Act as an anti‐regime mobilisation tool. The whole country became more sensitised towards what was happening in the scheme. The regime's claims that its agricultural policies would end poverty in Sudan were effectively debunked. Importantly, this was happening in Gezira state, which was considered to be one of the Islamists' strongholds. As such, it played a key role in turning public opinion against the regime.

In October 2017, a gathering organised by the Gezira Sons' Union decided to elect their own farmers' union despite the government's abolition of such bodies. Speakers emphasised how history was repeating itself, referring to the time before independence when farmers forced the colonial government to accept their union. Opposition political parties, other activists, and civil society organisations promised to provide their full support.^9^ By 2018, farmers' movements and the United Canabi Congress were part of the broad coalition of Forces of Freedom and Change (FFC) that led to the revolution that ousted President Al‐Bashir.

### 5.2 The transitional government exposes tensions in Al‐Gezira

Soon after the euphoria of ending 30 years of dictatorship, tensions between the different revolutionary forces became evident, reflecting competing interests. In addition, Prime Minister Abdalla Hamdok was unable to meet all of their demands and aspirations, and, worse, had to maintain a neoliberal regime that deepened the economic crisis. This subsection discusses each of these processes.

The FFC initially enjoyed the support of all revolutionary civil societies, resistance committees, and political parties, but entered into a power‐sharing agreement with the Transitional Military Council (TMC) that eventually undermined the revolution. The transitional government was composed of a Sovereign Council headed by TMC generals, notably, Abdel Fattah al‐Burhan of the SAF and Mohamed Hamdan Dagalo, known as Hemedti, of the RSF, and an Executive Cabinet headed by Hamdok.

The Hamdok government faced enormous revolutionary demands that it could not meet. In 2019, the Al‐Gezira Farmers' Alliance held a media conference and called for the immediate abolition of the 2005 Act and the 2014 amendments, all those involved in the scheme's collapse and deterioration to be held accountable, the irrigation system to be returned to a re‐established Gezira Board, and the establishment of a new fair and inclusive law. They insisted on improved standards of living, social and economic development for farmers, and government support for agriculture in general (and the scheme in particular) as a way of achieving food sovereignty. The United Canabi Congress also raised its demands, including the provision of a modern state of citizenship, without which people could not own agricultural land, and experienced discrimination and social humiliation. They also demanded the establishment of a federal commission on issues facing the Canabi, such as inadequate basic services and political representation on the basis of equal citizenship.

Hamdok took the controversial step of relying almost entirely on external support to ensure the availability of strategic goods such as wheat and fuel, while the military and RSF leaders directly controlled hundreds of companies (500 companies belonged to the military–industrial complex and 22 companies and economic corporations belonged to the RSF) (Alsilaik, 2023). Even the Central Bank of Sudan was under the control of military and paramilitary leaders of the Transitional Sovereignty Council, and it was not in their interest to support Hamdok (el‐Battahani, 2023). Worse, Hamdok's external funding was limited and conditional on extreme neoliberalist measures. Access to World Bank and IMF loans required the floating of the Sudanese pound on the international market and the lifting of bread and fuel subsidies (Baldo, 2021). The latter caused bread prices to increase from SDG 3 to 50, crucially undermining the transitional government's political legitimacy (Thomas and El Gizouli, 2020). These international institutions also suggested a targeted Family Support Program (FSP) to replace state subsidies, providing the equivalent of just USD 5 per person per month to 80 per cent of the population. In the end, it was only half‐funded and did not start until February 2021, covering 400,000 out of an intended 13.4 million beneficiaries (World Bank, 2021). As such, it was unable to mitigate the shock of the economic reforms. In 2021, 14.3 million people were estimated to be in need of humanitarian assistance (UN OCHA, 2021). These neoliberal reforms had a devastating political cost and contributed first to a military coup and then to war.

In relation to agriculture, Hamdok initially responded positively to the demands of the Al‐Gezira farmers, but tensions were exposed when he was not able to follow up with action. He abolished the 2005 Act, established a committee for a new 2021 Act, and appointed a President of the Gezira Board of Directors from among the intellectual leaders of the Farmers' Alliance. In addition, he announced a national campaign to rehabilitate and develop the Gezira scheme. He emphasised that this was a government priority, directing the local and foreign private sector to participate in upgrading its infrastructure (Al‐Democrati, 2021). However, the government's responses remained at a discursive level and structural causes of poverty and inequality were not tackled. Addressing farmers' demands was incompatible with the general liberalisation steps that the government had to follow. The Farmers' Alliance categorically rejected the draft 2021 Act, arguing that it contained all the defects of the 2005 Act and that it opened the door for international companies to buy Gezira land (Altaghyeer, 2021).

Meanwhile, the United Canabi Congress redirected its demands towards the Sudan Revolutionary Front (SRF; an alliance of rebel groups) and to the RSF, which were engaged in the Juba peace negotiations. This move was linked to the defection of the SRF from the FFC, a shift rooted in perceptions that the latter was not dealing with the root causes of war or demands to establish a state inclusive of all citizens. In practice, the Juba Peace Agreement (JPA), signed on 3 October 2020, did nothing to advance the cause of the Canabi people and the JPA signatories allied with the military component of the transitional government. JPA signatories prepared the political landscape and contributed to the coup of 2021, conceptualising it as ‘necessary corrective measures’ (SUNA, 2020).

The Canabi people failed to achieve the citizenship rights for which they longed and struggled and lost the negative peace that they “enjoyed” before the revolution. Their alignment with the military, the SRF, and the RSF was looked at suspiciously by their neighbours. They suspected that the Canabi would be the point of entry for RSF and Darfuri rebel movements to ‘peaceful’ Gezira land. This discontent materialised in some Al‐Gezira villagers attempting to remove forcefully their neighbouring labour camps, burning houses and properties in at least four of them (Radio Dabanga, 2020).

Some observers pointed the finger at old Islamist regime members for instrumentalising such deeply‐rooted cleavages as a way of further spoiling the transition and its peace processes (Radio Dabanga, 2020). What matters most, though, is that the tensions that were suppressed during Al‐Bashir's regime were exposed and led to violence between the different groups living in Al‐Gezira. This violence mirrored that in Darfur, Blue Nile, and eastern Sudan, where violence resumed or worsened following the JPA.

Most importantly, the transitional government was unable to bring the RSF's business empire and the SAF's military–industrial complex under its control. These predatory business networks have underpinned Sudan's conflicts and extractive political economy. In the past decade, their regional networks have expanded with the supply of soldiers to Saudi forces for the war in Yemen, control of gold mines, and livestock exports, with support from regional patrons like Egypt, Saudi Arabia, and the United Arab Emirates (UAE) (Hoffmann and Lanfranchi, 2023). A newly‐established anti‐corruption committee attempted to bring military–security‐owned companies under civilian control, coupled with security sector reform. But both General Burhan of the SAF and General Hemedti of the RSF resisted and launched a coup in October 2021. Resistance committees, opposition political parties, and civil society organisations, such as the Farmers' Alliance, never ceased protesting against this coup, despite internet shutdowns and heavy crackdowns. Development funding and loans were suspended (including the FSP), thus reducing even further the possibility that revolutionary demands and aspirations would be met. Sudanese people, including those in Gezira state, had been hard hit by Hamdok's mediated, but externally‐imposed, neoliberal economic measures, and negligible pledged support in the form of the FSP. The effect of these measures has been insufficiently recognised as contributing to the coup and deepening food insecurity even before the war.

### 5.3 The 2023 war, displacement, and the manipulation of pre‐existing tensions

The tensions and inequalities, between farmers, labourers, and Islamist companies, and the extractive political economy they represent, feed into the current (post‐2023) war. As discussed in the section on ‘war and famine in 2024’, the start of the war in April 2023 in Khartoum forced millions of people to flee the capital. Gezira represented a key destination for refuge because of its proximity to Khartoum and because it was one of Sudan's most food‐secure states. This subsection discusses what happened after this displacement and the invasion of the RSF later in the year, in relation to pre‐existing tensions and the extractive political economy.

Those displaced to Gezira followed different trajectories depending on their economic status and social connections to the state's population. Both Al‐Gezira villagers and Canabis or labourers received relatives who had long ago migrated to Khartoum as a way of coping with the deteriorating scheme and to pursue different livelihood strategies. This increased vulnerability to food insecurity among the local families who supported them. Cultivation was affected by the farmers' inability to access finance, and the lack of fuel for land preparations. Some farmers shifted cultivation to brown beans, locally known as *adasiya*, for food security rather than producing cash crops.^10^

The second category of IDPs were the ones who could rent apartments in the main urban towns. The third category were those without relatives or money and were accommodated in schools and mosques. They received some support from international and national organisations, local ERRs, and the Ministry of labour and Social Development. WFP set up one of its main warehouse hubs in Wad Madani, the capital of Gezira state, and by the end of 2023, was distributing food to about 800,000 people there (Al Jazeera, 2023). As elsewhere in the country, the ERRs were one of the most important aid providers, relying mostly on financial transfers from diaspora members, relatives, and friends to buy food and distribute it (Shabaka, 2023).

In December 2023, the RSF invaded Wad Madani, displacing hundreds of thousands of people, and disrupting production, trade, and aid. Those who were displaced from Khartoum were among the first to leave, along with farmers and labourers who fled when the RSF attacked their villages; they were then disconnected from the outside world owing to the internet blackout (starting in early February 2024) (Reuters, 2024). The occupation severely disrupted production as December–March is harvest time. The RSF enforced its presence on farms during the harvest so that it could subsequently loot it.^11^ More importantly, the RSF controlled every aspect of production and food supply—warehouses, seeds, fertiliser, pesticide, and irrigation canals—giving it the means of forcible conscription by threatening denial of food (Munsi et al., 2024). WFP and other international organisations fled, and their warehouses were looted (Al Jazeera, 2023).

Once the RSF controlled the area, only RSF‐linked traders could function. The number of merchants in what are now RSF areas has dramatically declined.^12^ Some wholesalers were able to source fertilisers and agricultural inputs from east Sudan; however, they face key challenges in terms of access to finance and insecurity (Hoffman, Lanfranchi, and Alhag, 2024). Going forward, the provision of inputs and finance to merchants in Al‐Gezira will be vital to production and to how the political economy evolves. In the past, companies supplying agricultural inputs and services were closely linked to the Islamist regime. Many of these will have left. During the transitional government, though, large family corporations and small and medium‐sized enterprises were able to grow, and policy analysts suggest that aid organisations support them to mitigate the threat of famine (Hoffman and Lanfranchi, 2023). At the same time, there is a risk that multinational corporations, perhaps linked to Egypt or the UAE, take over land and production in Al‐Gezira (thereby controlling a large part of the national political economy). As seen in earlier sections, Egypt and the UAE have played critical roles in supporting the RSF or the SAF. Politically, control of Al‐Gezira is important, as it strengthens negotiating positions in any future peace talks.

The risk of external land grabs in Al‐Gezira has increased with the RSF's occupation. During the war, the Ministry of Finance pledged all Gezira scheme assets (worth approximately USD 3 billion) to the Agricultural Bank of Sudan in exchange for financing the winter season, which was eventually destroyed by the RSF. This means that the fate of farmers, and the scheme, is insolvency, and might result in the comprehensive seizure of land and assets by creditors, ‘zeroing’ the scheme's book value for the next buyer.^13^ A meeting between the Minister of Finance and Economic Planning and the Gezira scheme delegation highlighted the importance of introducing large foreign capital and extensive experience to achieve a radical change in the project (Al Hadath, 2024).^14^ Others argue that the RSF's occupation of Al‐Gezira, with its base of Arab nomadic pastoralists, could be seen as righting the historical wrong of expulsion from its original rangelands.^15^ While this has not yet appeared in its discourse, or been reflected in its governance of the state, the RSF may be using a similar strategy to that in parts of Darfur: (re)turning the land to pastoralism and livestock production for export to the Gulf states (Duffield and Stockton, 2023).

With movement of material aid becoming almost impossible in Sudan, digital technologies, including cash transfers, have become a crucial way of providing assistance (for international and local non‐governmental organisations and ERRs). Aid and agricultural and other public services all require connectivity to be able to function. For this reason, communications systems have been weaponised, continuing Sudan's long history of aid manipulation. From early February, the RSF disabled all internet providers in Sudan (Al Jazeera, 2024; Reuters, 2024). This crucially affects any financial transfers; digital transfer through the Bankak app is one of the only ways of moving money—this has halted aid operations (Amnesty International, 2024; Islamic Relief Worldwide, 2024). Even the local ERRs have had to stop their activities. This not only further increases the risk of starvation, but it also feeds into the war economy. One of the only ways to be connected now is to use Starlink satellites (Siddig, 2024), which ordinary people pay to use—brought in and managed by the RSF from early 2024. As such, control over communications has become a way of denying services and resources to the enemy, maintaining control over life‐saving communications and finance for ordinary citizens, and a new way of profiteering. The SAF has banned the use of Starlink.

The RSF–SAF war is also feeding tensions between farmers and labourers. When the RSF was advancing, the SAF and related security forces were already accusing the (Canabi) labourers of being potential RSF allies, leading to some of them being killed. The RSF in turn used these accusations as part of its war propaganda to show that these were SAF racist manifestations of the 1956 state that it is fighting to remove. If the Canabi formally align themselves with the RSF, this will have disastrous consequences. At least when there was a negative peace, they could produce food. Now there is a risk of violent confrontation between labourers and farmers and production will stop, leading to potentially the most apocalyptic chapter of the Al‐Gezira story. The Canabi constitute at least half of the population, and the way that they are geographically distributed—every village has a neighbouring camp—means that there will be war everywhere in Gezira state. Since February 2024, some Canabi joined the RSF willingly, others were forcibly conscripted, and some resisted. This is the case with the farmers as well. There is polarisation, but not as clear‐cut as two groups opposing each other in war. Whether this will descend into an even uglier civil war or de‐escalate needs to be closely observed. It is inextricably linked to the national process of war and peace.

What is happening in Sudan in 2024 is the manifestation of an intensifying extractive political economy (Duffield and Stockton, 2023). Without addressing issues of equal citizenship, it will not be possible to reduce the tensions between the Canabi and farmers in a meaningful manner. And that means that divisions can continue to be manipulated by the warring parties to escalate the war, and an inclusive political economy would not be achieved. In the coming months and years, it will be important to continue to research these issues in Sudan.

## 6 DISCUSSION AND CONCLUSIONS

This article has shown how food insecurity is intimately connected with historical political and economic processes, using the example of the Gezira agricultural scheme and its role in Sudan's political economy overall. Neoliberalisation measures featured prominently in these processes, implemented at different points in time since the 1980s, impoverishing farmers and pitting tenants against labourers, but enabling those close to the former regime to benefit. These tensions and inequalities continue to play out today, in early 2024, feeding into the war and its disastrous consequences in terms of displacement, famine, and death.

The political economy of neoliberally‐motivated processes has been evident in the self‐serving problematisation of Gezira scheme management, resulting in the dissolving of the Gezira Board, the liquidation of the scheme's publicly‐owned assets, and the privatisation of agricultural service provision. This led to deteriorating infrastructure, eroded the collective bargaining power of farmers, and increased vulnerability to exploitation and inequalities. Together these different power relations formed the structural causes of poverty and food insecurity for farmers and labourers. The failure of the Gezira scheme was used by anti‐regime movements to call for revolution, but the post‐revolution government adopted even more severe neoliberal measures, dissipating its political legitimacy because of the resulting economic and food crisis. The transitional government was unable to meet the demands of farmers and labourers in Al‐Gezira, for a fair and inclusive agricultural scheme and equal citizenship; this helped to pave the way for the 2021 military coup and a deteriorating economic and food security situation. From 2023, existing animosities resulted in direct violence and displacement. When the RSF invaded Al‐Gezira, both the RSF and the SAF manipulated the existing tensions between tenants and labourers as part of their war strategy. This tactic, coupled with mismanaged international and regional peace interventions, have (always) led to elitist power‐sharing agreements rather than an inclusive dialogue resulting in a democratic transition (Assal, Abdul‐Jalil, and Egemi, 2020).

Sudan provides an example of how decades of war, neoliberal economic strategies, and inequality combine to feed into, or create, a highly extractive political economy. As suggested by Duffield and Stockton (2023), the de‐development this results in can be seen as capitalism's last food frontier. With the war entering Al‐Gezira, and with Gulf states or regional actors supporting either the RSF or the SAF, fertile agricultural land in Sudan is literally being fought over by national and international actors. As much of the country's population is already at risk of famine, it presents an extension of the ‘benefits of famine’ through violent or market‐mediated asset transfer (Duffield, 1994; Keen, 1994). Furthermore, Sudan, as one of capitalism's last frontiers, should not only be seen in terms of food, but also digital technologies, financial transfers, and data (now the world's most valuable commodity). Like food aid before, connectivity has been weaponised by the warring parties, with shutdowns and private provision in certain areas. For cash transfers, aid organisations also work with digital and financial service providers, merchants, and banks. While at present this kind of innovation is urgently needed to supply life‐saving services, the dangers of capitalism without a state should not be underestimated. Such a country would be unlikely to provide protection or welfare to its most vulnerable or marginalised citizens, instead turning them into a flexible and exploitable labour force. In Sudan, these would be people such as the labourers on the Gezira scheme, or the displaced from Darfur and the Nuba Mountains—often one and the same. An analysis of political economy or power relations is rarely incorporated in food security assessments or the evaluations of aid programmes (Jaspars, 2018).

This article provides an argument for including a deeper historical understanding of power dynamics in the more common technocratic picture of food security, to understand fully what causes vulnerability and the actions that are needed to address it.

## ETHICS STATEMENT

This paper reports analysis of primary data. The ethics of data collection and analysis were approved by the SOAS Research Ethics Panel.

## FUNDING STATEMENT

We would like to acknowledge the African Peacebuilding Network of the Social Science Research Council, for generously funding the ethnographic fieldwork carried out in 2017–18. In addition, we would like to thank the Economic and Social Research Council for providing the funding for the ongoing research project on ‘Digitalising Food Assistance’ (grant number: ES/X005550/1).

## Data Availability

Research data are not shared.^16^

## ENDNOTES

^1^
An Economic and Social Research Council‐funded project titled ‘Digitalising food assistance: political economy, governance, and food security effects across the global North–South divide’.

^2^
War started in Darfur in 2003. Three million people were still displaced, mostly in camps, prior to 2023. Power relations had already shifted following the revolution (in 2019) and the military coup (in October 2021), but violence became even more extreme from April 2023 when the RSF and allied militia forces could attack and destroy with impunity.

^3^
Observation by our research assistant.

^4^
Ethnographic information gathered by Tamer Abd Elkreem.

^5^
Interview with a leading member of the Farmers' Alliance.

^6^
Interview, 2017.

^7^
Field observation.

^8^
See https://www.youtube.com/watch?v=sk2LQK6BNNE (last accessed on 4 October 2024).

^9^
Tamer Abd Elkreem was present at the conference.

^10^
Observation by our research assistant.

^11^
Observation by our research assistant.

^12^
Interview with a company representative, 2024.

^13^
See https://www.facebook.com/aldaribsudan (last accessed on 19 September 2024).

^14^
See https://www.facebook.com/share/p/nqQ22btnJrY7L5Xv/?mibextid=xfxF2i (last accessed on 19 September 2024).

^15^
Personal communication with Nick Stockton. 2024.
